# The impact of prior endoscopic or surgical therapy on open Zenker’s diverticulum surgery: analysis on a large single center cohort: Comparison of primary and revisional open surgery for Zenker’s diverticulum

**DOI:** 10.1007/s00464-022-09690-0

**Published:** 2022-10-31

**Authors:** Rebekka Dimpel, Alissa Jell, Daniel Reim, Maximilian Berlet, Michael Kranzfelder, Thomas Vogel, Helmut Friess, Hubertus Feussner, Dirk Wilhelm

**Affiliations:** 1 Department of Surgery, Faculty of Medicine, Klinikum rechts der Isar, Technical University of Munich, Ismaninger Str. 22, 81675 Munich, Germany; 2 MITI Research Group (Minimally Invasive Interdisciplinary Therapeutical Interventions), Klinikum rechts der Isar, Technical University of Munich, Munich, Germany

**Keywords:** Zenker’s diverticulum, Open diverticulectomy, Endoluminal treatment, Revisional surgery, Risk factor

## Abstract

**Background:**

Endoscopic treatment of Zenker’s diverticulum is an attractive minimally invasive alternative compared to the classic open approach. However, increased recurrence rate were reported. In case of relapse, endoscopic therapy might be repeated, or alternatively open surgery is performed. This study aimed to identify potential differences in the outcomes between primary or secondary surgical treatment in Zenker’s diverticulum.

**Methods:**

From January 2003 to April 2019, 227 subsequent patients underwent surgical diverticulectomy and cervical myotomy at the surgical department of TUM. 41 of 227 patients had received previous therapy, either open or endoscopic. Perioperative parameters in priorly untreated patients were retrospectively compared to those after previous therapy (mostly endoscopic) with special regard to perioperative data and postoperative complications. Univariate and multivariate regression analyses were performed to identify predictors for postoperative complications.

**Results:**

We could show that the number of complications (*p* = 0.047) in pretreated patients is significant higher as well as the severity after Clavien–Dindo (*p* = 0.025). Stapler line leakage, wound infections, and operative revision rate was higher also pretreated group. Pretreatment and surgery time showed a significant association with postoperative complications in univariate analysis. In multivariate analysis, pretreatment remained a significant independent predictor of complications.

**Conclusion:**

The present data indicate that endoscopic therapy might represent a risk factor for postoperative complications in case of relapse surgery. Therefore primary open surgery should be debated in patients with an increased high risk of relapse.

**Graphical abstract:**

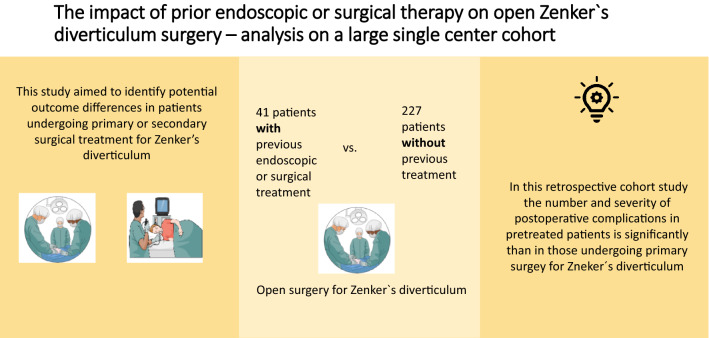

Zenker’s diverticulum is a disease of the aged population and in recent times preferentially treated by an endoscopic approach. In comparison to the conventional, open technique, the endoscopic therapy is proven to be advantageous in terms of shorter hospitalization, reduced morbidity and an almost zero mortality rate [1–3]. Zenker’s diverticulum is a rare disease which is affecting only 2 out of 100.000 patients, and only few studies regarding outcomes of endoscopic and open surgical treatments were published in recent years [4–6]. So far, no randomized studies comparing the two therapeutic approaches exist. The most comprehensive analysis was published by Verdonck et al. as a systematic review who identified a failure rate of 18.4% for endoscopic and 4.2% for the open approaches. In this study the corresponding complication rate for endoscopic treatment was 7% and 11% for open surgery. Accordingly, one of the authors’ conclusions was that the open technique is more successful but causes much more complications [4].

Evidence for recurrence rates of Zenker’s diverticulum after previous treatments is sparse and so far only isolated publications on small case series on re-do endoscopic interventions were published. Here, the authors describe the efficacy and safety of the endoscopic method. Due to the good and low complication feasibility of endoscopic therapy, patients often undergo multiple endoscopic therapies in case of recurrence [2, 3, 7]. Thus, most of the patients being referred for open surgical repair, have had previous endoscopic treatments in an increasing percentage. This leads to the notion that open surgery is considered a second line alternative and only indicated as an “if all else has failed” solution. To our knowledge, only two retrospective studies addressed the question before whether endoscopic interventions influence the outcome of open surgery in case of recurrence [7, 8]. In comparison, our study has a higher number of patients and compares primary operated patients with patients operated in case of recurrence. The aim of this retrospective study was to analyze whether previous treatment might have an influence on complication rates and the clinical outcomes surgery for Zenker’s diverticulum.

## Materials and methods

The medical records of all patients (*N* = 227) treated surgically for Zenker’s diverticulum at the department of surgery, Klinikum rechts der Isar, from January 2003 to April 2019 were retrospectively analyzed. A database (Microsoft Excel, Redmond, WA, USA) including the size of diverticulum, operative time, length of hospital stay, therapy related complications, pre-existing conditions, demographic variables and number of endoscopic diverticulostomies was established and perioperative parameters in respect of endoscopic pretreatment were retrospectively analyzed.

Patients were followed-up for 30 days after the operation or for the duration of hospital stay when patients were hospitalized longer.

Patients without previous treatment (primary surgery) were allocated to group A.

Comparison group B represents patients who have had either one or more flexible endoscopic myotomy or rigid endoscopic treatment or primary surgery and underwent open surgery for recurrence.

Descriptive statistics on patient characteristics were calculated as the mean ± standard deviation (continuous variables), and frequencies (categorical variables). Categorical variables were compared using either the chi-square test or Fisher’s exact test as appropriate.

The factors for postoperative complications were analyzed by uni- and multivariable regression analyses. Variables assessed in these terms were: size according to the Brombart classification, age, gender, pretreatment, diabetes, reflux, ASA classification, anticoagulation, operative time. After univariable analysis only statistically significant variables were entered in the multivariable model. Results of the logistic regression analyses were reported with odds ratio (OR) together with the corresponding 95% confidence intervals (CI).

Statistical analyses were performed using SPSS version 25 (IBM Inc., Ehningen, Germany). *p* values less than 0.05 were considered statistically significant. This retrospective analysis was approved by the local IRB (No 801/20 S-SR; Ethikkommission der Fakultät für Medizin, TUM School of Medicine).

### Operative technique

All operations were performed under general anesthesia and standardized by an experienced surgeon or under his assistant.

After left cervical incision and identification of the esophagus, a flexible tube (36Ch) was perorally inserted into the esophagus under external manual guidance for exposure and protection. The diverticulum was then isolated and the muscle fibers of the cricopharyngeal muscle completely dissected. Subsequently, the diverticulum was resected with linear stapler application (Covidien TA 45 blue cartridge). In case of a very small diverticulum (Brombart I and II) only a diverticulopexy was performed with fixation of the base of the diverticulum superiorly to the prevertebral fascia. Finally, a drainage tube was inserted into the wound and the wound closed in layers.

The standard procedure for recurrent Zenker diverticulum is the same as for primary surgery.

## Results

### Study population

From January 2003 to April 2019, 227 patients underwent open treatment for Zenker’s diverticulum at the department of surgery of TUM. All patients received a comprehensive preoperative evaluation in a standardized fashion: barium esophagogram, gastroscopy, additional esophageal manometry and pH-metry in almost all patients. In all patients the Zenker’s diverticulum was confirmed by fluoroscopy and the indication for surgery was established upon clinical associated symptoms, such like dysphagia, weight loss, regurgitation, and aspiration.

### Patient characteristics

186 patients received open surgery (group A), 41 received surgery for recurrence after previous treatment (group B). In this group, 1–8 prior interventions were registered, with 21 patients having undergone a single flexible endoscopic septotomy, 3 patients having passed 2 endoscopic interventions, 2 having undergone 3 endoscopic interventions and one patient 8 endoscopic dissections. In 6 patients received rigid endoscopy and 7 patients underwent primary open pretreatment.

The largest proportion of previously treated patients received treatment outside our clinic, only 3 patients 73.2% (3/41) received primary therapy in our clinic.

There were no significant differences between groups (A/B) for gender, age, pre-existing conditions, immunosuppression, and general comorbidity assessment according to the ASA classification (Table 1). Also, the size of Zenker’s diverticulum at time of surgery according to the Brombart classification showed no significant differences (*p* = 0.483) and was classified as Brombart III or IV in most patients. Furthermore, the age distribution of patients was typical for the disease and averaged around 70 years in both groups. As potential influencing factor we also evaluated the intake of anticoagulants such like aminosalycylates or clopidogrel and cumarines. No difference was seen between groups either (*p* = 0.23).

**Table 1:** Baseline characteristics

	Patients with pretreatment (*N* = 41)		Patients without pretreatment (*N* = 186)		*p* value
	*n*	%	*n*	%	
Female	17	41.46	56	30.11	0.196
Male	24	58.54	130	69.89	
Diabetes	3	7.32	8	4.30	0.423
No diabetes	38	92.68	178	95.70	
Reflux	4	9.76	22	11.83	1
No reflux	37	90.24	164	88.17	
Immunosuppression	1	2.44	4	2.15	1
No immunosuppression	40	97.56	182	97.85	
ASA classification					
1	4	9.76	26	13.98	0.597
2	29	70.73	126	67.74	
3	7	17.07	33	17.74	
4	1	2.44	1	0.54	
Brombart					
1	0	0.00	3	1.61	0.483
2	2	4.88	13	6.99	
3	10	24.39	61	32.80	
4	29	70.73	109	58.60	
No anticoagulation	35	85.37	161	86.56	0.805
Anticoagulation	6	14.63	25	13.44	
Ass	1	2.44	15	8.06	0.230
Marcumar	5	12.20	8	4.30	
Plavix	0	0.00	1	0.54	
Xerelto	0	0.00	1	0.54	
Age in mean		69.80	67.25		0.155

Two patients suffering from previously not diagnosed squamous cell carcinoma, which was found in the Zenker diverticulum after resection.

## Perioperative results

To investigate perioperative differences, operative time, complication rates according to Clavien–Dindo and the required therapy for complication management, as well as the duration of postoperative hospitalization were analyzed.

The following variables were included in the univariable regression analysis: Brombart, age, gender, pretreatment, diabetes, reflux, ASA classification, anticoagulation, operative time, and immunosuppression, because these are the most relevant factors to predict postoperative complications. Univariable regression analysis revealed pretreatment (*p* = 0.006) and surgical procedure time (0.0032) as significantly associated with postoperative complications (Table 2). Only the significant variables from the univariable regression analysis were entered in the multivariable model. The multivariable analysis demonstrated only pretreatment (*p* = 0.01) as a significantly and independent risk factor (Table 3).

**Table 2:** Univariable regression analysis

	OR	CI%95 lower	CI%95 upper	*p* value
Brombart	0.916	0.552	1.520	0.734
Age	0.995	0.964	1.028	0.771
Gender	0.934	0.453	1.925	0.853
Pretreatment	2.926	1.368	6.259	**0.006**
Diabetes mellitus	0.978	0.203	4.701	0.978
Immunosuppression	1.104	0.120	10.135	0.931
Reflux	1.055	0.374	2.981	0.919
ASA classification	0.687	0.388	1.216	0.198
Anticoagulation	0.603	0.249	1.463	0.263
Surgery time	0.981	0.964	0.998	**0.032**

Statistically significant values (*p* < 0.05) is given in bold

*OR* odds ratio, *CI95*% *lower* 95% confidence interval lower boundary, *CI95*% *upper* 95% confidence interval upper boundary, *ASA* American Society of Anaethesiologists

**Table 3:** Multivariable regression analysis

	OR	CI%95 lower	CI%95 upper	*p* value
Pretreatment	2.64	1.22	5.71	**0.01**
Surgery time	0.98	0.97	1.001	0.07

Statistically significant value (*p* < 0.05) is given in bold

*OR* odds ratio, *CI95*% *lower* 95% confidence interval lower boundary, *CI95*% *upper* 95% confidence interval upper boundary

The mean operative time showed significant differences between groups and was 46.6 min (range 20–151 min) in the primary surgery group compared to 54.17 min (range 27–83 min) in the pre-treatment group (*p* = 0.005). Median postoperative hospital stay was 7 days in both groups. (3–41 days for group A and 5–35 days for group, *p* = 0.098). Shown in Table 4.

**Table 4:** Complications rates and postoperative results for patients with and without pretreatment

	Patients with pretreatment (*N* = 41)		Patients without pretreatment (*N* = 186)			*p* value
	*n*	%	*n*		%	
No complications	27	65.85	158		84.95	0.063
Abscess	2	4.88	6		3.23	
Wound healing disorder	3	7.32	4		2.15	
Suture insufficiency	3	7.32	6		3.23	
Others	6	14.63	12		6.45	
Nervus recurrens palsis	3	7.32	7		3.76	0.392
No palsis	38	92.68	179		96.24	
Bleeding	1	2.44	3		1.61	
Surgical revision	2	4.88	7		3.76	0.667
Surgery time in mean	54.17	46.63				0.005
Hospital stay in median	7 [5–35]	7 [3–41]				0.062

Specific complications in detail were as follows: The stapler line leakage rate was 7.32% (3/41) in patients who had been treated before compared to 3.23% (6/186) in the primary group. Comparable results were detected for the number of surgical site infections (7.32% (3/41) versus 2.15% (4/186). The most serious complication was observed in one patient in the pre-treated group who developed a mediastinitis following fistulation of the stapler line. Postoperative bleeding occurred in 1 patient (2.44%, 1/41) in the pretreated group and two (1.61%, 2/186) in non-pretreated group.

Surgical revision rate was higher in patients that had undergone prior treatment (2/41 4.88%) compared to the primary surgery group (7/186, 3.76%), (*p* = 0.667).

In the group of pre-treated patients, 7.32% (7/41) developed recurrent laryngeal nerve palsies postoperatively, compared to 3.76% (7/186) of patients in the primary surgery group (*p* = 0.392).

Complication grades according to Clavien–Dindo are shown in Table 5. There was a significant difference in the frequency of the respective CD-grades between both groups (*p* = 0.047). Regarding the severity of complications according to Clavien–Dindo in the two studied groups, it was shown that in the group of pretreated patients there were significantly more severe complications (Clavien–Dindo III/IV) than in the primary surgery group (*p* = 0.025).

**Table 5:** Complication table for Clavien–Dindo: complications subdivided by severity according to Clavien–Dindo, the complications were I and II, and III and IV was summarized, respectively

	No complications		CD I/II		CD III/IV		*p* value
	*n*	%	*n*	%	*n*	%	
Patients with pretreatment (*N* = 41)	27	65.84	9	21.95	5	12.20	0.025
Patients without pretreatment (*N* = 186)	158	84.95	16	8.60	12	6.45	
	185	81.50	25	11.01	17	7.49	

*CD* Clavien–Dindo

In correlation to the respective pre-treatment patterns (one endoscopic pretreatment, multiple endoscopic pretreatments, and/or surgical open pretreatment), it was shown that patients with multiple endoscopic pretreatments had significantly more postoperative complications grade I/II (5/12, 41.67%) and grade III/IV (4/12, 33.33%) than surgically pretreated and non-pretreated patients.

## Discussion

This retrospective study to date investigated the largest cohort of patients having undergone open surgery for Zenker’s diverticulum in a single institution and is one out of only three studies that examined the influence of endoscopic pre-treatment on subsequent open surgery. However, the existing literature on recurrence has small patient numbers concludes that both endoscopic and open therapy are feasible in case of recurrence [7, 8].

Based on this retrospective analysis of 227 patients having undergone surgery for Zenker’s diverticulum it was shown that pretreatments might have a significant influence on the occurrence of postoperative complications. In univariable regression analysis it was shown that operating time, which could be regarded as a surrogate parameter for the complexity of an intervention, serves as another significant factor for the development of postoperative complications. In addition, we were able to show that significantly more severe complications according to Clavien–Dindo occur more frequently in pretreated patients.

Zenker’s diverticulum is a disease of the old who observe progressive dysphagia and sometimes also a bulge of the left cervical silhouette. Treatment is increasingly becoming the domain of interventional endoscopy and flexible septotomy. This is mainly due to the known advantages of endoscopic treatment as shorter in-patient stay, reduced invasiveness and low complication rates [1, 2, 9–11].

These are clear arguments in favor of the endoscopic procedure and against open surgery. Past reviews comparing open and endoscopic procedures in terms of complication rates show a higher complication rate for the open procedure with 11%. They demonstrated stapler line leakages of 4.7%, recurrent laryngeal nerve injury rates of 5.7% and pneumonia rates of 2.8% [4, 5]. However, and often neglected, endoscopic treatment is associated with a significantly higher recurrence rate and also reduced effectiveness in symptom control [12]. Protagonists of endoluminal treatment consider these drawbacks as being of lesser importance due to the ease of repeating the procedure if required. Up to now, however, it is still unclear how often redo myotomy can or should be repeated and whether open surgery in first line is really outdated [7].

Relapses after primarily successful interventions are always disappointing both for the patient as well as for the physician, even if the procedure can be performed easily and safely again. One previous study investigated 25 patients with recurrent Zenker’s diverticulum after an endoscopic and open approach, which they compared to 34 consecutive primary cases. All patients received endoscopic flexible septum division. In the group of patients with recurrent Zenker’s diverticulum complication rates were nearly similar to the primary group (8 vs. 8.8%). But 28% of patients with recurrent Zenker’s diverticulum required more than one treatment and the relapse rate in the pre-treated group accounted for 24% compared to 14,7% in the primary endoscopic treatment group [8].

Here, the advocates of endoscopic treatment point to the studies which have shown that reinterventions with an endoscopic approach are safe and technically feasible [7]. On the other hand, voices are increasingly raised that in particular young patients have an increased risk of recurrence of a Zenker’s diverticulum after endoscopic therapy [13], and, on the other hand, have a longer remaining life span. It has also been shown that open surgery has lower recurrence rates, especially in the recurrence situation [2, 12, 13]. Of course, it must not be forgotten that the majority of patients demonstrate high comorbidity rates and that there is a clear advantage here with regard to endoscopy in terms of the lower post-interventional complications [14].

From the surgeon’s point of view it is known that redo procedures are usually associated with a higher complication rate than primary procedures. This distinctive difference may be explained by clinically relevant micro- and macroscopical tissue alterations. Scar formation is inevitable after tissue dissection, even after primary wound healing. After endoscopic diverticulostomy, clinical signs of microperforation such as cervical or mediastinal emphysema occur in about 10% of cases and the incidence of clinically inapparent bacterial inflammation is certainly even higher [15]. This makes the identification of anatomical dissection layers more demanding than in primary surgery and explains the prolongation of the surgery and the higher incidence of complications. Healing of surgical sutures demands adequate tissue perfusion and tension free adaptation of the intestinal wall, both of which can be impaired by prior intervention, no matter if done endoscopically or in an open way.

The extent to which endoscopic pretreatment alters the tissue for subsequent surgery is not quantifiable. Although endoscopic treatment takes place only intraluminally, the tissue appears to be more adhesive compared to patients who had not undergone endoscopic pretreatment.

It may be speculated that endoscopic pretreatments provoke adhesions because in this procedure the septum is split and a common channel is created that must be dissected, separated, and then closed.

This assumption is supported by the results of the present study. Although there were no statistically significant differences in the need for reoperations, the rate is higher in the pre-treated group. Furthermore, the abscess rates, suture incidence and wound healing problems were significantly higher in the pre-treated group.

The study has several limitations. It is a retrospective analysis of prospectively documented patients. There is a considerable heterogeneity in the pretreated group. A potential bias in the analysis of the present patient cohort is that the expertise in our center is mainly in open surgery, which is related to the fact that the true denominator of endoscopic treatments and the true success is not known because mostly recurrences were referred to our center. It could be that the endoscopic therapy is highly successful and only those patients with a clinical problem were seen in our center. Majority of patients underwent flexible endoscopic pretreatment, with a small proportion receiving rigid endoscopy or open surgery. If only endoscopically pretreated patients are compared to the non-pretreated patients, too few events occur that would allow for a meaningful regression analysis to determine independent prognostic factors in regard of complications. However, in subgroup analysis in which once endoscopically flexible, rigidly pretreated, multiple endoscopically flexible and rigidly pretreated patients and openly preoperated patients were included, there was a significant difference in the number of complications and in the severity scores according to Clavien–Dindo. Due to the extremely small number of patients resulting from the subgroup analysis conclusive statements may not be taken. Furthermore, we only assessed patients for the first 30 days postoperatively, as patients are in general returned to the family doctor for subsequent treatment soon after surgery. Accordingly, we cannot make any statement on the long-term outcomes of these patients. Besides this, dysphagia scores were not recorded to address functional outcome, as this was not the main focus of this investigation.

The present data does not allow to determine whether endoscopic or open surgical treatment is the better approach for Zenker’s diverticulum, but we would like to raise awareness that endoscopic pretreatment might have an influence on the development of postoperative complications, if open surgery deems necessary and that the preference for endoscopic treatment is not based on reliable data or on randomized controlled prospective data. Especially in smaller Zenker diverticula (Brombart I and II), flexible endoscopic threshold splitting has its limitations because in most cases, complete transection of the horizontal pars of the cricopharyngeal muscle cannot be achieved [6]. It remains speculative if endoscopic treatment alone might be the reason for increased complication rates in recurrent Zenker’s surgery. Conclusively, this analysis points out that pre-treatment has an influence on open revision surgery. Therefore, especially younger patients have to receive special information. It is suggested that the endoscopist must inform the patient about possibly higher complication rates if in the long run open surgery is required.
